# Rehabilitation of atrophic jaw using iliac onlay bone graft combined with dental implants

**DOI:** 10.1186/s40729-019-0163-9

**Published:** 2019-03-19

**Authors:** Truc Thi Hoang Nguyen, Mi Young Eo, Tae Seong Kuk, Hoon Myoung, Soung Min Kim

**Affiliations:** Department of Oral and Maxillofacial Surgery, Dental Research Institute, School of Dentistry, Seoul National University, 101 Daehak-ro, Jongno-gu, 03080 Seoul, South Korea

**Keywords:** Dental implant, Iliac bone grafting (IBG), Marginal bone loss (MBL), Survival rates

## Abstract

**Background:**

Rehabilitating severely atrophic alveolar crests remains challenging for implantologists and maxillofacial surgeons. Recently, a combination of augmentation and dental implantation has been used to treat cases with severe bone atrophy in the maxilla and mandible. Among autogenous bone grafts, iliac bone grafting (IBG) is considered safe for collecting large amounts of bone and obtaining high-density multipotent cells. However, vertical bone resorption may occur during the initial healing stage after IBG. The purpose of the present study is to evaluate bone graft success and implant survival rate, along with bone height in the augmented site and marginal bone level around dental implants placed in iliac onlay bone grafts. We also introduce technique and treatment considerations for successful IBG procedures, as well as optimal implant installation strategy and soft tissue manipulation.

**Methods:**

We examined seven patients who were treated with IBG combined with implant systems over a period of 10 years. The long-term success rate of bone grafts and implant survival rate were recorded. Bone height change and marginal bone loss (MBL) were analyzed by assessing the radiograms acquired after augmentation, at implant installation, prosthetic loading, and after installation 1 year, 2 years, 3 years, and 5 years.

**Results:**

In a mean observation period of 50 months (range 12–62 months), the success rate of IBG was 100%. A total of 29 implants were installed and the implant success rate was 100%. The mean bone height reductions compared to post-augmentation bone heights were 1.33 ± 0.81 mm after 3 months, 2.00 ± 1.88 mm at implant installation, 2.55 ± 1.68 mm at prosthetic loading, and 3.05 ± 1.63 mm after implant installation 1 year. The cumulative bone height change after implant installation 5 years was 4.05 ± 1.83 mm which corresponds to a mean resorption rate of 42.5%. The mean MBL after installation 3 months, at prosthetic loading, and after installation 1 year, 2 years, 3 years, and 5 years follow-ups were significantly higher than at implant installation. However, MBL at 2 years, 3 years, and 5 years post-installation did not differ significantly (*p* < 0.05).

**Conclusion:**

In patients with atrophic jaws, a combination of the iliac onlay bone graft and dental implants can result in satisfactory reconstruction and reliable long-term prognosis. Despite early stage vertical bone resorption, we observed high success rates and comparable MBL over long-term follow-up. To reduce bone resorption, case evaluation and surgical planning must be meticulous. Further large-scale studies with longer-term follow-up are needed.

## Background

Over the past decade, dental implants have become a routine treatment for the rehabilitation of partially and totally edentulous patients [1]. Sufficient residual bone volume is essential for the retention and stability of implants and to achieve favorable outcomes. In many cases, dental implants can be placed without any obstructions; however, in some cases, unfavorable local conditions such as jaw atrophy, bone defects due to diverse types of osteomyelitis, cancer ablation surgery, and trauma sequelae may result in insufficient bone volume for implant installation due to defects in one or multiple dimensions. These situations are also associated with the proximity of anatomical structures, such as the inferior alveolar nerve, the maxillary sinus floor, or the nasal floor, which all complicate the ability to provide adequate implant therapy.

Atrophied alveolar crest cases may be treated using short dental implant (SDI) of which length is less than 8 mm [2–4]. Several other techniques are also currently being used to treat bone deficiency when the use of SDI is impossible due to severe bone defects, including guided bone regeneration techniques, autogenous bone graft (ABG), alveolar distraction osteogenesis, and vascularized free flap bone reconstruction [5].

Among these surgical techniques, ABGs have advantages in atrophic jaw augmentation for dental implant installation [6, 7]. There are several intraoral and extraoral donor resources for ABG. Intraoral bone can be harvested from the maxilla and mandible, including the mandibular symphysis and parasymphysis (chin region), the mandibular ramus, maxillary tuberosity, and coronoid process. Intraoral bone blocks have low resorption rates but are not always available and sometimes lack adequate volume for grafting [8, 9]. In cases with large bone defects, extraoral donor sites are necessary. Extraoral donor bone can come from the iliac crest, calvarium, or tibia [5, 10]. In recent years, iliac bone graft (IBG) has become one of the most commonly used ABG technique for a wide spectrum of indications and are considered reliable in cases of large bone defects [11].

The clinical results reported for reconstructed bone success and implant survival in IBG reconstruction are comparable to those of other ABG techniques [11, 12]. However, the long-term survival rate and stability of reconstructed bone in IBG are still controversial due to uncertainties regarding bone resorption rates, especially during the early healing period [13, 14].

The purpose of this study was to evaluate bone graft success and implant survival rates, along with bone height in augmented sites and marginal bone loss (MBL) around dental implants placed in iliac onlay bone grafts. We examined a total of seven patients who were treated with IBG in combination with implant systems over a period of 10 years. All patient radiological data were analyzed to determine bone height changes and MBL during follow-up. We discuss tips regarding technique and treatment to facilitate successful IBG, optimal implant installation strategy, and soft tissue manipulation.

## Methods

This study is reported following the Strengthening the Reporting of Observational Studies in Epidemiology (STROBE) guidelines [15]. Patients included in this retrospective study were treated at Seoul National University Dental Hospital, Seoul, Korea between December 2008 and April 2018. Records for a total of seven patients with 29 dental implants (Straumann® Dental Implant System, Institute Straumann AG, Basel, Switzerland) were analyzed clinically and radiologically. The study protocol and access to patient records were approved by the Institutional Review Board of Seoul National University, Seoul, Korea.

Patient records were retrospectively reviewed to identify patients that fulfilled the following inclusion criteria:

1. Age > 18
2. Lack of medical conditions contraindicating IBG surgery and implant surgery
3. Insufficient residual bone volume for implant installation or achievement of primary implant stability without any bone regeneration or augmentation
4. Receipt of iliac bone onlay graft
5. Treatment with a two-stage surgical protocol
6. Implant treatment using bone level (BL) or tissue level (TL) dental implant (Straumann® Dental Implant System, Institute Straumann AG, Basel, Switzerland)
7. Fabrication and delivery of prosthesis following implant installation
8. Clinical and radiogram data available for all treatment periods and follow-up visits

Patients with IBG follow-up periods of less than 6 months or lacking data regarding bone graft or implant installation were not included in the analysis.

### Surgical procedures

**Fig. 1:**
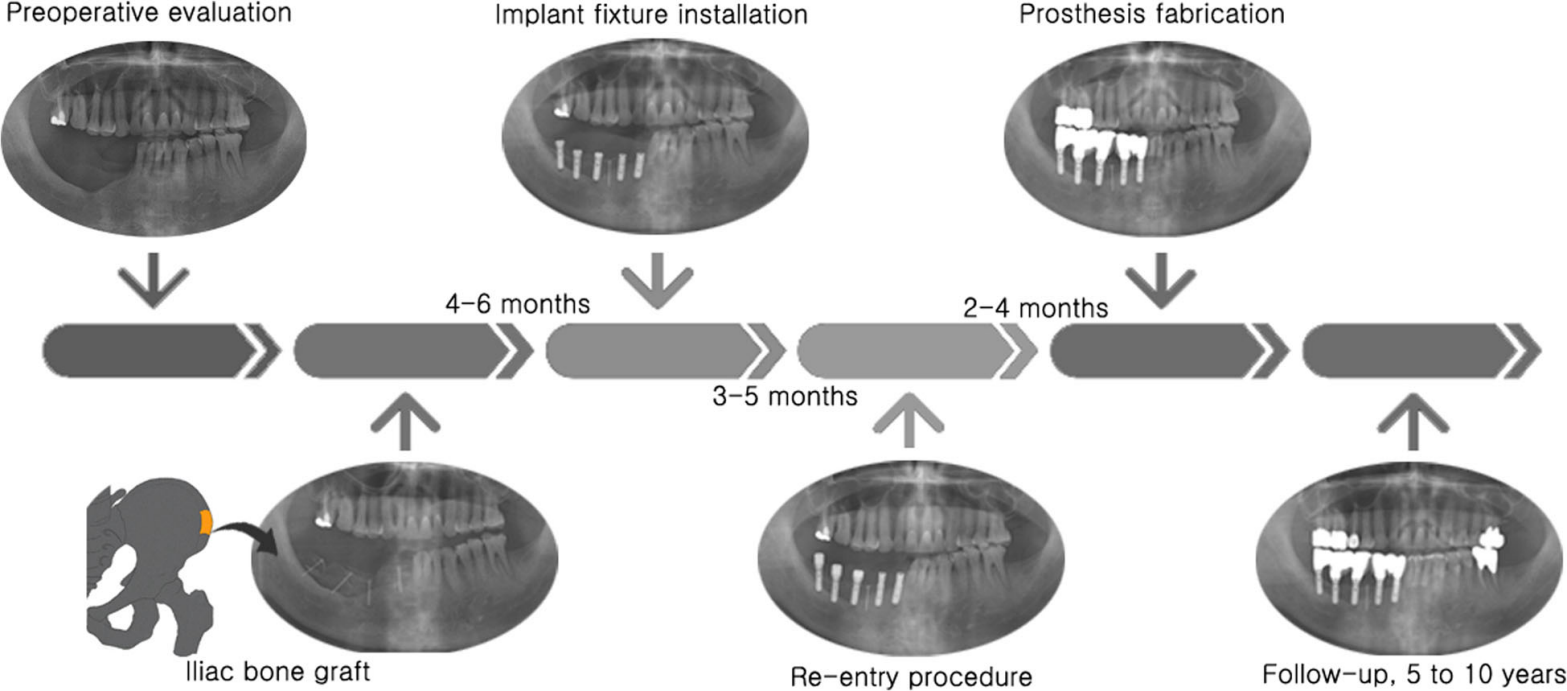
Schematic representation of the treatment protocol for iliac bone grafting and implant installation, including bone block harvesting from the iliac crest on lower left drawing

**Fig. 2:**
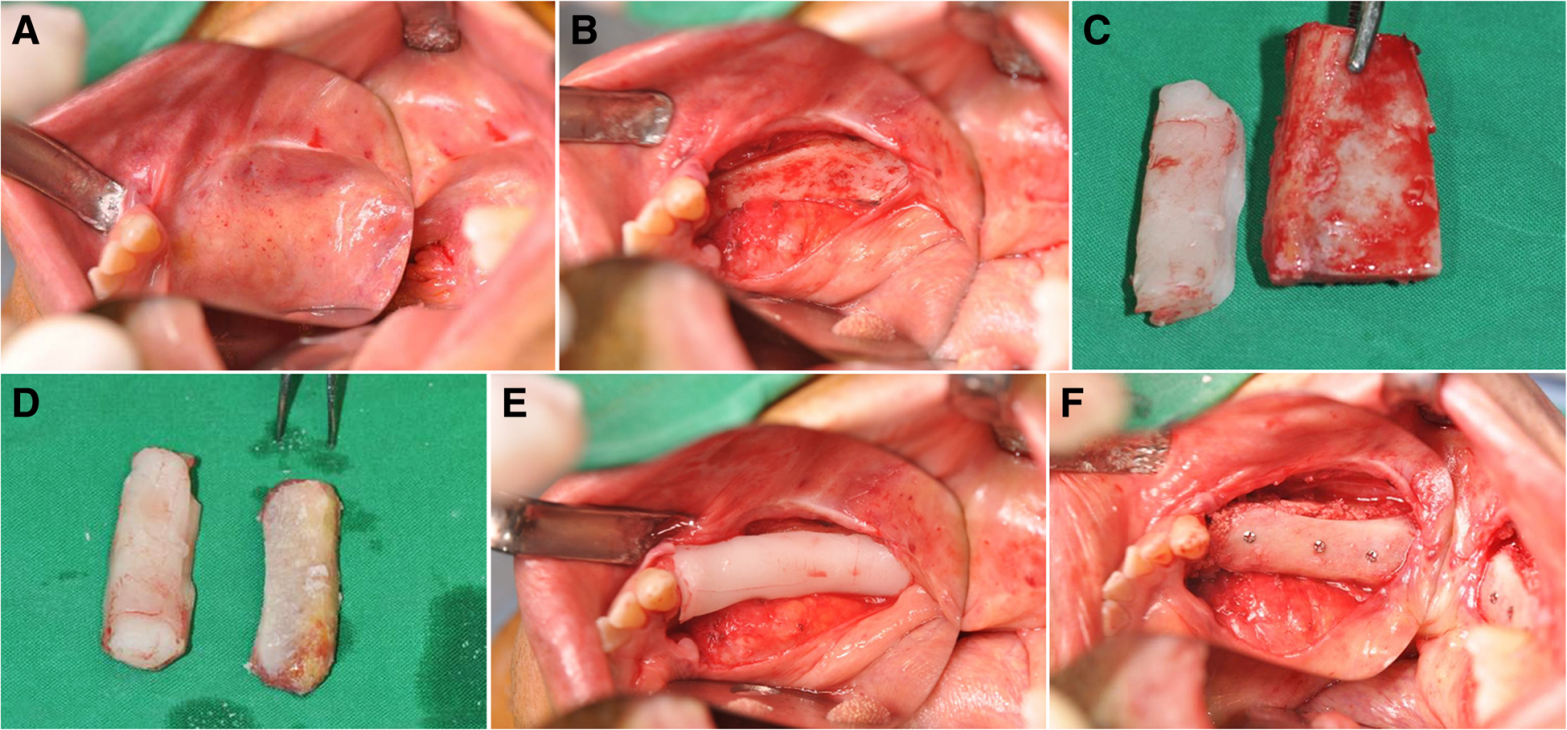
Clinical views of patient no. 07 during the bone graft stage showing pre-operative view of the right edentulous posterior region of the mandible (**a**), elevation of a full thickness mucoperiosteal flap to expose the deficient edentulous area (**b**), the iliac bone block was modeled to obtain precise adaptation to the recipient region (**c**, **d**), and iliac bone block was fixed to the basal bone using titanium microscrews (**e**, **f**). All gaps between bone blocks were filled with autogenous particulate bone

The surgical procedure included two stages: reconstructive surgery and implant installation surgery performed 4 to 6 months after reconstruction (Fig. 1). All surgical procedures (bone reconstructions and implant installation) were performed by one surgical team. Iliac bone harvesting and ridge reconstruction were performed continuously under general anesthesia. After being harvested, the iliac bone blocks were modeled to achieve the precise size and shape of the recipient site. In the deficient edentulous area, a full-thickness flap was elevated and the bone blocks were then fixed to the basal bone by titanium microscrews. The remained bone was processed to make particulate bone that was used to fill gaps between the iliac bone blocks and the original bone. After the completion of all reconstructive procedures, release incisions were made to obtain tension-free closure of the surgical flaps (Fig. 2).

### Implant installation stage

Implant length and diameter were chosen base on the bone volume gained from the bone augmentation in the implant sites and base on the prosthetic indication. Implant design (bone level (BL) or tissue level (TL)) was decided according to the gingival biotype analysis result.

**Fig. 3:**
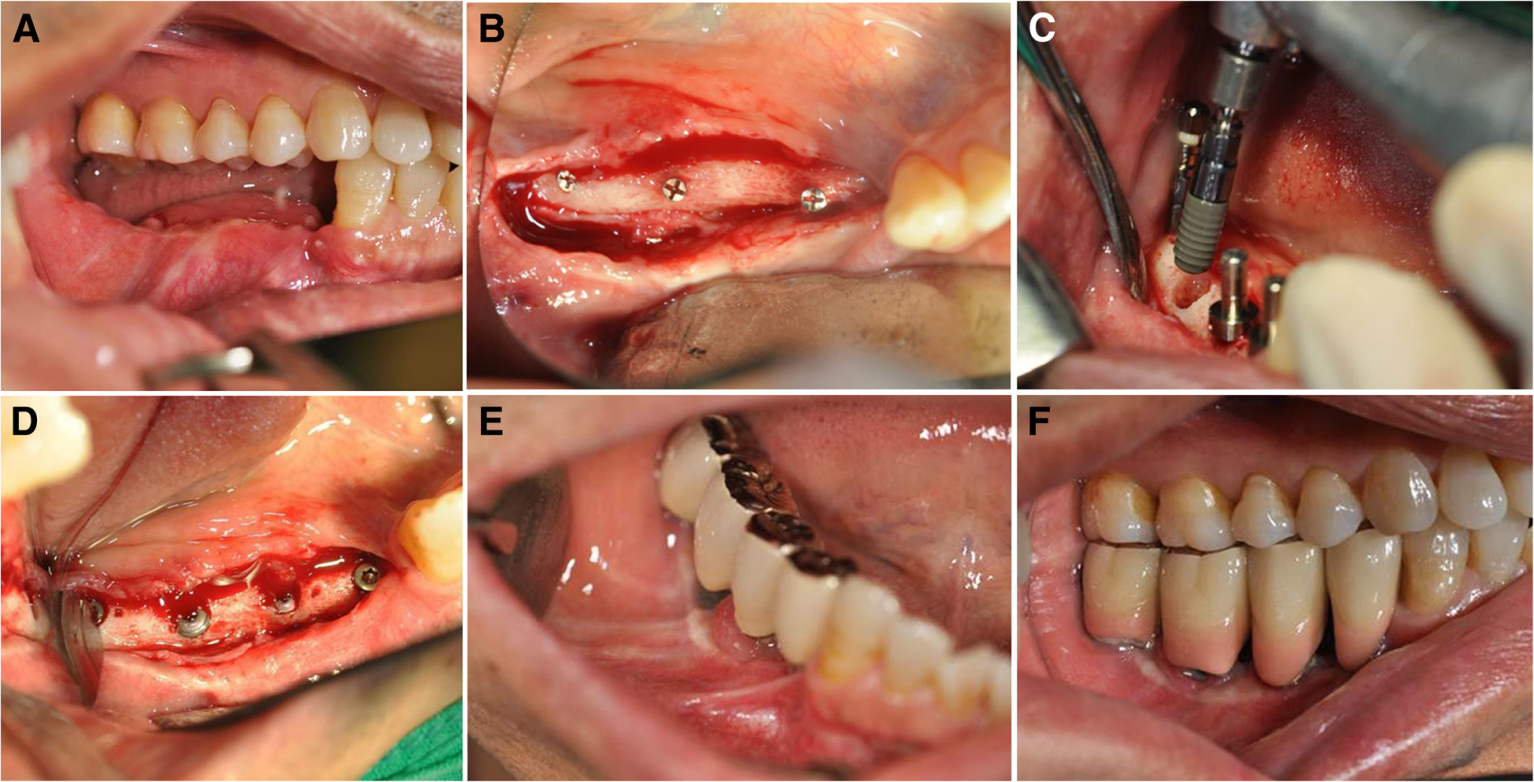
Clinical views of patient no. 07 during implant installation and prosthesis fabrication showing grafted site after 6 months healing (**a**), second stage surgery for screw removal (**b**) and implant fixtures installation (**c**, **d**), and final prosthesis fabrication (**e**, **f**)

A full-thickness flap was elevated using the same incision line for conventional implant placement. Then, the microscrews used to fix the grafts were removed. When a screw was difficult to approach and was not interfering with implant positioning, the screw was left in place to avoid any unnecessary graft exposure or bone invasion. Both BL and TL implants (Straumann® Dental Implant System, Institute Straumann AG, Basel, Switzerland) were placed following the instructions of the manufacturers. The BL implants were inserted with up to the same level of crestal bone. Meanwhile, the TE implants were inserted with a smooth neck section of 2.8 mm above the bone level. Primary stability was achieved in every case. After implant insertion, the cover screw was connected (Fig. 3). The mucoperiosteal flap was carefully sutured to submerge the BL implants. In the TL implants, a non-submerged method was used and the soft tissue was sutured tightly around the implant neck. We tried to keep the retained attached gingival zone on the deficient alveolar ridge. This goal was also emphasized in the re-entry procedure.

### Prosthetic fabrication

Prosthetic fabrication was carried out 3 to 5 months following implant insertion in TL implant. In BL implants, 3 to 5 months after installation surgery, the re-entry surgery was performed. The cover screws were removed and changed into healing abutments. After the second implant surgery, once proper conditioning of the soft tissues was obtained, the prostheses were fabricated and delivered (Fig. 3).

### Implant and prosthetic maintenance

At-home implant and prosthetic care played an essential role in long-term maintenance. Patients were introduced to the adequate oral hygiene control method using soft toothbrush, interproximal brush, and silk floss or hydro floss in the treatment process and were re-educated on each recall visit.

The follow-up visits were scheduled every 3 months in the first year after loading, every 6 months in the second year, and were scheduled once every following year if there was no abnormal condition and patient oral hygiene was well controlled. In old patients or patients with poor oral hygiene practicing, follow-up visits were scheduled every 3 months. In each follow-up visit, the patient’s medical and dental history were updated as well as the oral hygiene condition. Clinical examination was performed to evaluate peri-implant and periodontal tissue, implant stability, and plaque index. Radiograph was used to detect any bony abnormality and evaluate alveolar bone around each implant. Scaling and curettage using plastic instruments were carried out if necessary.

### Evaluation parameter

The following parameters were registered and evaluated: (1) graft success rate, (2) implant survival rate, (3) augmented bone height change over time, and (4) MBL over time.

#### Graft success/failure evaluation

The success or failure of each bone graft was recorded according to Barone criteria [16]. A graft was considered successful if there was no graft exposure during healing, no infection, and no radiolucency on radiograph examinations. In addition, the graft had to achieve integration and immobilization at the recipient site, bleeding from the bone graft during drilling for implant placement, and adequate bone formation for implant insertion [16].

#### Implant survival evaluation

The implant failure criteria was based on the ICOI Pisa Consensus implant quality of health scale [17]. An implant was considered to have failed (clinical or absolute failure) if it had any of the following conditions: pain on function, mobility, radiographic bone loss > 1/2 the length of the implant, uncontrolled exudate, or was no longer in the mouth [17].

#### Bone height evaluation

Presurgical and postsurgical radiologic assessments were performed by panoramic radiogram to evaluate the bone height changes in graft sites and the MBL of implants. The images were grouped based on the following time points: after grafting surgery (within 3 days, Tg); 3 months after grafting surgery (Th); after implant installation (Ti); 3 months after implant installation (T1); after prosthesis loading (T2); and 1 year, 2 years, 3 years, and 5 years after implant installation (T3, T4, T5, T6, respectively).

**Fig. 4:**
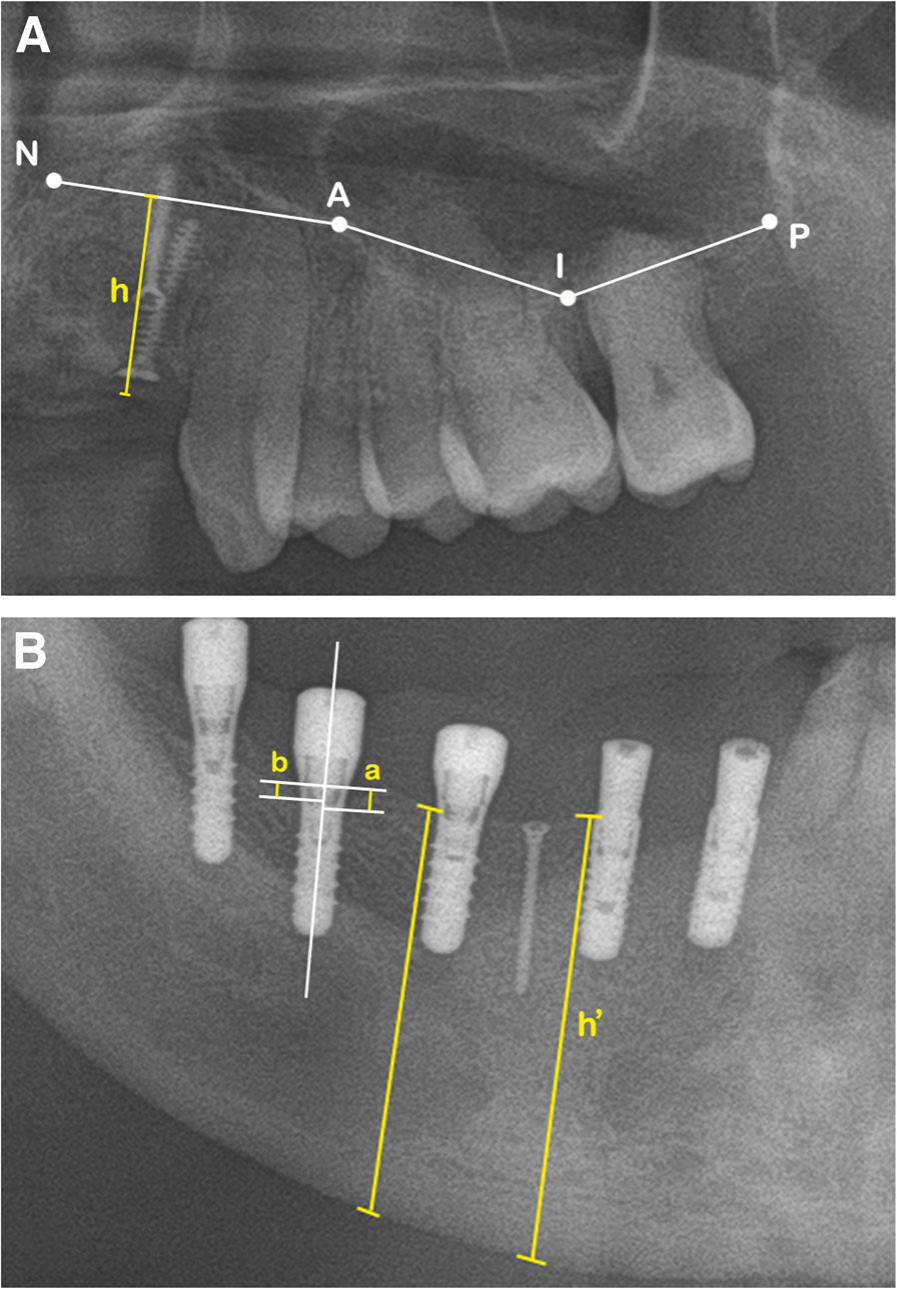
Panoramic view shows the reference points and lines used for bone height and MBL measurements. **A** In the upper jaw, N is the lowest point of nasal floor, and A and P correspond to the lowest parts of the mesial wall and distal walls, respectively. Point I is the median point between points A and P. h is the bone height, determined by the distance between the alveolar ridge to the upper limit. **B** h’ is the bone height, determined by the distance between the alveolar ridge and the border of the jaw; a and b are the mesial and distal MBL, respectively, determined by the distances from the most mesial and distal points of the implant platform to the crestal bone

In the upper jaw, bone height was measured according to Cawood and Howell [18]. The upper limits were the nasal floor and the maxillary sinus floor. On the panoramic radiographs, this upper limit was determined by connection of 4 points: N, A, I, and P. N was defined at the lowest part of the nasal floor. We used 3 points as standard points on the maxillary sinus floor: points A and P correspond to the lower parts of the mesial and distal walls, respectively, and point I is the median point between points A and P [19] (Fig. 4a). In the lower jaw, it was difficult to follow Cawood and Howell [18] because some patients had already undergone treatment for severe osteomyelitis or mandibular cancer. Therefore, the boundaries between the alveolar bone ridge and basal bone were hard to determine, and we instead measured bone height by drawing a line according to the implant axis. The distance between the alveolar ridge and the border of the jaw was recorded (Fig. 4b).

#### MBL evaluation

To determine MBL, we used linear measurements from the most mesial and distal points of the implant platform to the crestal bone (Fig. 4b). Average MBL of each implant was calculated from the mesial and distal MBL. MBL was measured on each panoramic radiograph by a single examiner (T.H.T.) and confirmed by other authors. Each site was measured three times and made as average value. All radiograms were corrected to the same magnification. All measurements were obtained using the PACS calibration system (PiView-Star®, version 5.0.1, Infinitt Co., Seoul, Korea).

### Statistical analysis

We collected both descriptive and quantitative data. Descriptive statistics were used to calculate and analyze means and standard deviations (SDs) of bone height loss and MBL. The Shapiro–Wilk test was used to test the normality of variables. If the data were normally distributed, the differences between follow-up periods were tested by paired Student’s *t* test. All analyses were carried out using SPSS (SPSS 25.0®; SPSS Software Company, Chicago, IL, USA). *p* values < 0.05 were considered statistically significant.

## Results

### Patients

Seven patients treated at the Department of Oral and Maxillofacial Surgery, Seoul National University, with iliac-only bone grafting were included in this study. Three patients were male, and four patients were female. The average patient age was 65 years (range 46–80 years) (Table 1). Among the seven patients treated with iliac onlay bone grafts, three patients were treated for severe atrophic conditions and four patients received bone augmentation after osteomyelitis surgery or marginal mandibulectomy. Two among the seven patients were totally edentulous, while the remaining five patients were partially edentulous.

**Table 1:** Patient and treatment data

Patient no.	Age/gender	Augmentation site	Number of implants	Prosthesis
1	80/F	Mandible	4	Hybrid denture
2	73/M	Mandible	5	Bridge
3	63/F	Maxilla, mandible	4	Bridge
4	46/M	Maxilla, mandible	6	Hybrid denture
5	70/M	Mandible	4	Overdenture
6	68/F	Mandible	2	Overdenture
7	55/M	Mandible	4	Bridge

### Graft success/failure evaluation

The graft success rate was 100% at the time of implant placement and at the last follow-up visit according to criteria for success defined by Barone et al. [16]. No postoperative complications or donor site complications were reported.

### Implant survival evaluation

After a mean healing period of 4 to 6 months, a total of 29 dental implants were installed in the augmented bone. Twenty-three implants were inserted in the mandible and six implants were installed in the maxilla. Fifteen implants were placed in the anterior region and fourteen implants were placed in the posterior region. One patient lost one implant before the second stage surgery, which was replaced by another implant in proximity to the original site. Using the ICOI Pisa implant quality of health scale, the survival rate of implants in the IBGs was 96.56% (Table 2).

**Table 2:** Implant survival rate

Augmented site	Success/fail (total)	Survival rate (%)
Maxilla	6/0 (6)	100
Mandible	22/1 (23)	95.65
Total	28/1 (29)	96.55

### Bone height evaluation

**Fig. 5:**
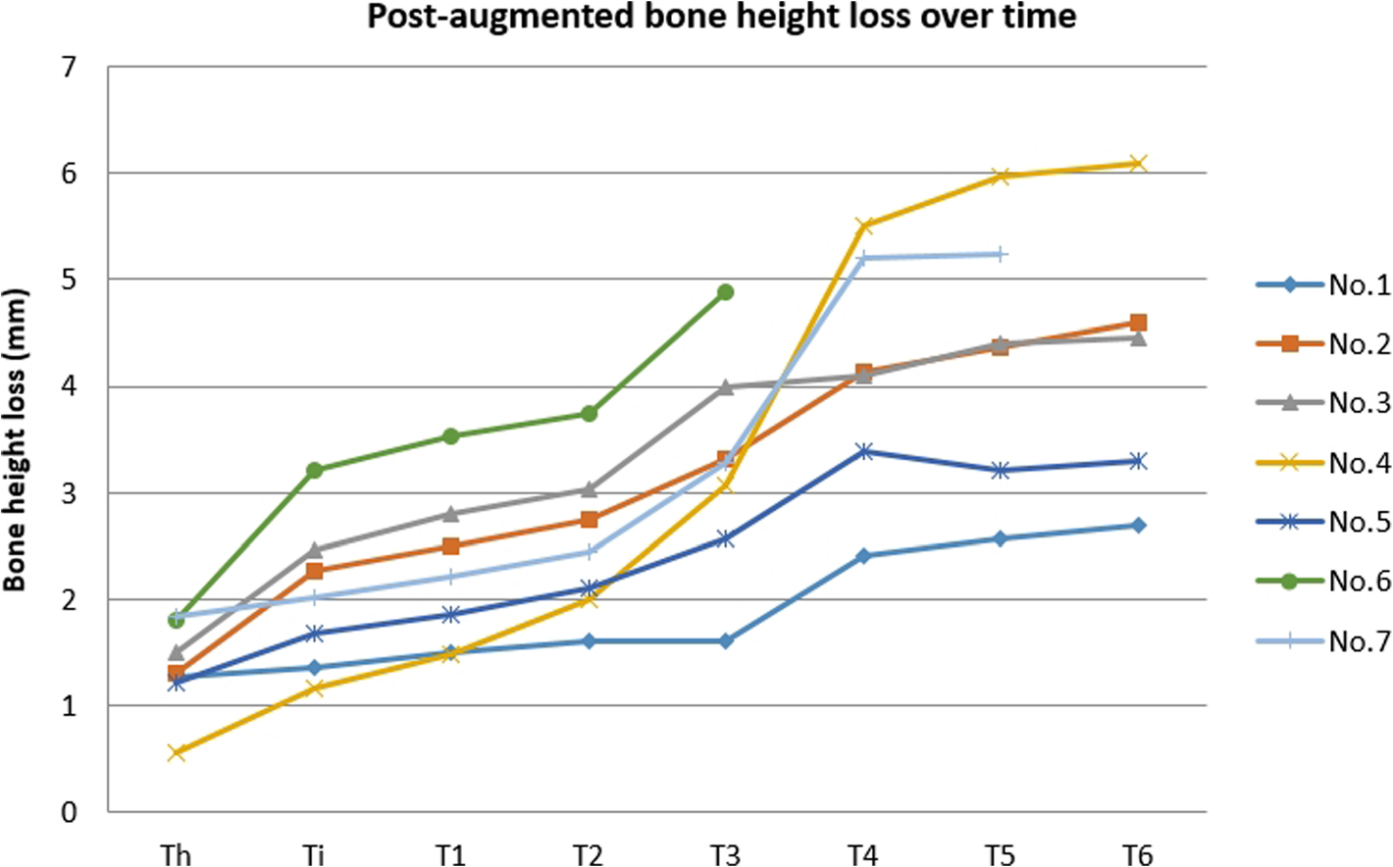
Mean bone height loss over time in individual patients. Th: 3 months post-augmentation, Ti: at implant installation, T1: 3 months after installation, T2: at prosthetic loading, T3: 1 year after installation, T4: 2 years after installation, T5: 3 years after installation, T6: 5 years after installation. In each patient, bone height changes showed high rates of bone resorption during the early period and in the first year after implant installation. Upon further follow-up, the bone height loss 3 years and 5 years post-installation did not differ significantly (*p* < 0.05)

After IBG surgery, the mean vertical bone gain was 9.53 ± 4.78 mm in the augmented sites. Three months after augmentation surgery, the mean bone height loss was 1.33 ± 0.81 mm (Th), which corresponds to a mean resorption rate (MRR) of 14%. The mean bone height loss was 2.00 ± 1.88 mm at implant installation (Ti), 2.29 ± 1.70 mm at 3 months post-installation (T1), and 2.55 ± 1.68 mm at prosthetic loading (T2), which corresponds to MRRs of 21%, 24%, and 26.8%, respectively. After 1 year (T3), 2 years (T4), and 3 years (T5) post-installation, the mean bone height loss values were 3.05 ± 1.63 mm, 4.02 ± 1.5 mm, and 4.12 ± 1.82 mm, respectively, which corresponds to MRRs of 32%, 42.2%, and 43.2%, respectively. The mean cumulative bone height loss at 5 years post-installation (T6) was 4.05 ± 1.83 mm, which corresponds to an MRR of 42.5%. The bone height loss at Th and Ti differed significantly. Also, bone height loss at T2 and T3, T3, and T4 differed significantly, respectively. Bone height loss at T4, T5, and T6 did not differ significantly (*p* < 0.05) (Table 3). In each patient, bone height change measurements showed high rates of bone resorption in the early period and in the first year after implant installation. In further follow-up, bone height loss at 2 years, 3 years, and 5 years post-installation did not differ significantly (Fig. 5).

**Table 3:** Mean bone height (BH) loss and mean resorption rate over time

	Mean BH loss (mm)*	Mean resorption rate (%)**
Th	1.33 ± 0.81	14%
Ti	2.00 ± 1.88	21%
T1	2.29 ± 1.70	24%
T2	2.55 ± 1.68	26.8%
T3	3.05 ± 1.63	32%
T4	4.02 ± 1.50	42.2%
T5	4.12 ± 1.82	43.2%
T6	4.05 ± 1.83	42.5%

Th: 3 months post-augmentation, Ti: at implant installation, T1: 3 months after installation, T2: at prosthetic loading, T3: 1 year after installation, T4: 2 years after installation, T5: 3 years after installation, T6: 5 years after installation

*BH at Th and Ti differed significantly. BH at T2 and T3, and T3 and T4 differed significantly. BH at T4, T5, and T6 did not differ significantly (*p* < 0.05)

**Mean bone height gained after augmentation was 9.53 ± 4.78 mm

### MBL evaluation

**Fig. 6:**
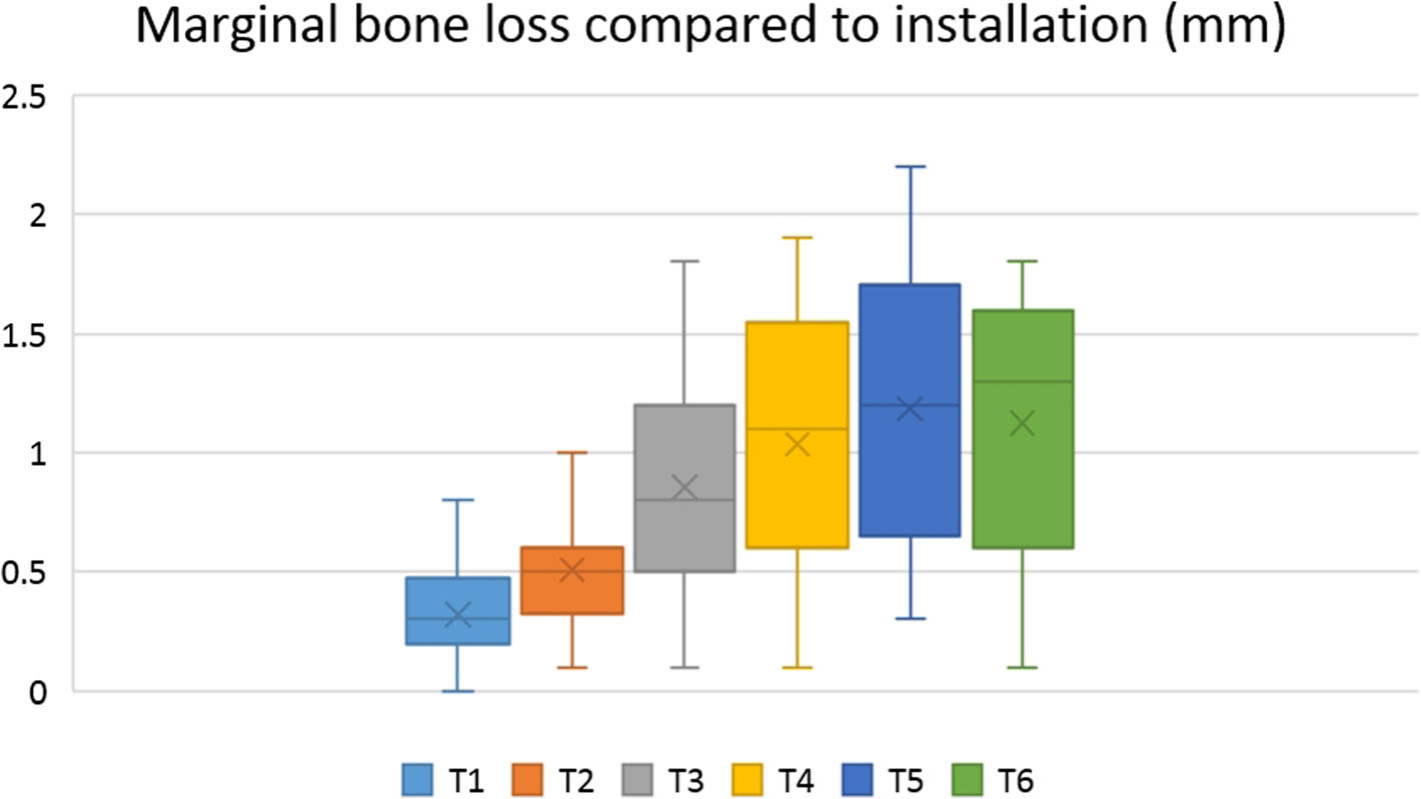
Marginal bone loss (MBL) for each follow-up period compared to MBL at installation. T1: 3 months after installation, T2: at prosthetic loading, T3: 1 year after installation, T4: 2 years after installation, T5: 3 years after installation, T6: 5 years after installation. Mean MBL at 3 months, prosthetic loading, and 1 year, 2 years, 3 years, and 5 years after installation was significantly higher than MBL at the time of implant installation (*p* < 0.05)

**Fig. 7:**
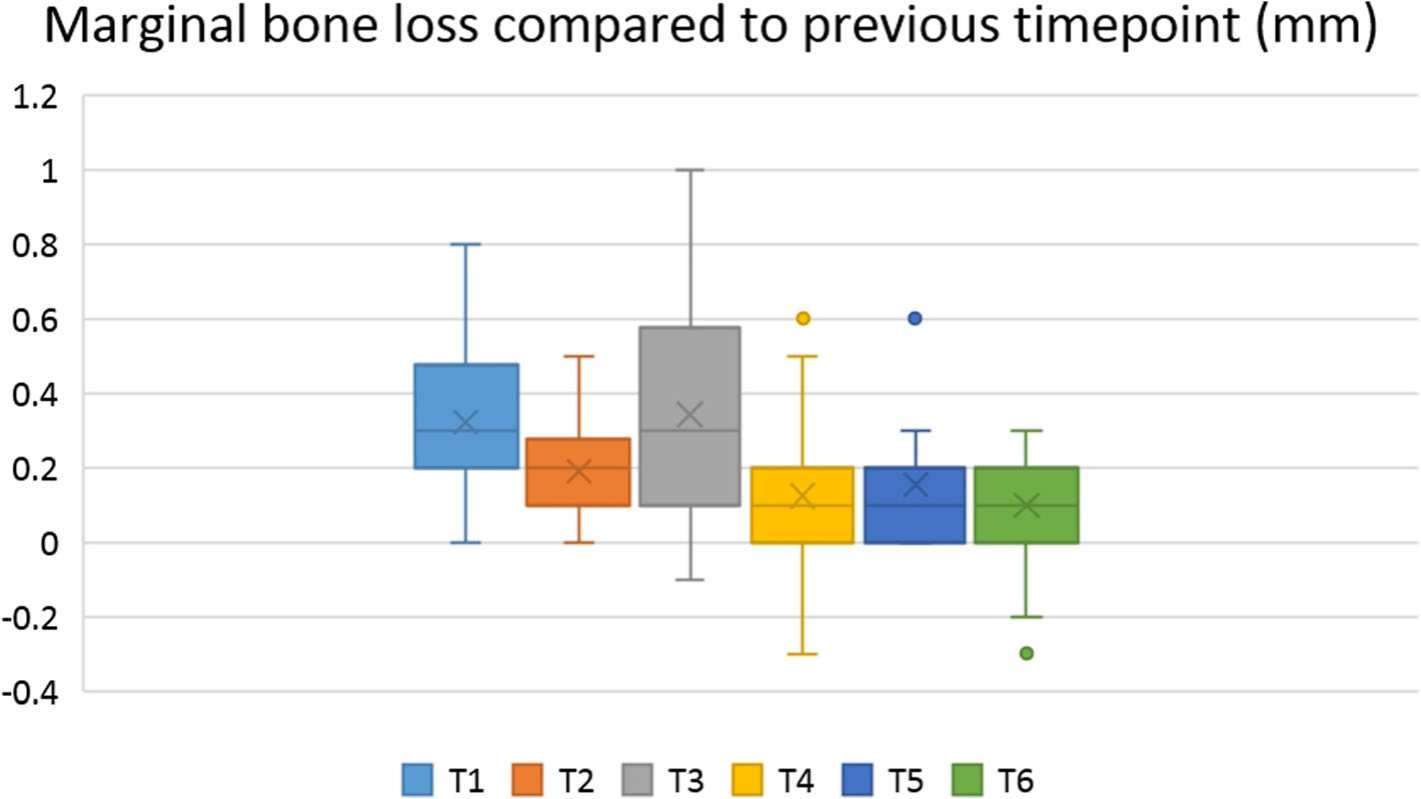
Marginal bone loss at each time point compared to MBL at the previous visit. 3 months after installation, T2: at prosthetic loading, T3: 1 year after installation, T4: 2 years after installation, T5: 3 years after installation, T6: 5 years after installation. Mean MBL change compared to previous time point significantly differed between T2 and T1, T3 and T2, and T4 and T3. MBL change at T4, T5, and T6 did not differ significantly (*p* < 0.05)

The MBL values at each time point were compared to MBL at the time of installation. MBL measured at each time point also was compared to previous MBL values (Table 4). Mean MBL values at 3 months post-installation, prosthetic loading, and 1 year, 2 years, 3 years, and 5 years after installation were significantly higher than at the time of implant installation (*p* < 0.05) (Fig. 6). Furthermore, mean MBL at prosthetic loading was significantly higher than at 3 months post-installation, and mean MBL at 1 year after installation was significantly greater than at prosthetic loading. However, MBL change after 2 years, 3 years, and 5 years post-installation did not differ significantly (*p* < 0.05) (Fig. 7).

**Table 4:** Marginal bone loss (MBL) of dental implants placed on the augmented bone at evaluated time points

	MBL compared to installation (mm)*	MBL compared to previous time point (mm)**
T1	0.32 ± 0.21	0.32 ± 0.21
T2	0.51 ± 0.24	0.19 ± 0.14
T3	0.85 ± 0.44	0.34 ± 0.29
T4	1.03 ± 0.53	0.12 ± 0.20
T5	1.19 ± 0.57	0.15 ± 0.18
T6	1.12 ± 0.50	0.10 ± 0.16

T1: 3 months after installation, T2: at prosthetic loading, T3: 1 year after installation, T4: 2 years after installation, T5:3 years after installation, T6: 5 years after installation

*Mean MBL at T1, T2, T3, and T4 was significantly higher than at implant installation (*p* < 0.05)

**Mean MBL change compared to previous time point significantly differed between T2 and T1, T3 and T2, and T4 and T3. MBL change at T4, T5, and T6 was not differ significantly (*p* < 0.05)

There were three groups regarding prosthetic indication, including bridge, hybrid denture, and overdenture. The prosthetic indication was made according to each patient clinical condition in order to achieve the highest function and esthetic. The difference between MBL value at 5 years post-installation and MBL at prosthetic loading of the bridge group was 0.60 ± 0.31 mm, of the hybrid denture group was 0.64 ± 0.36 mm, and of the overdenture group was 0.58 ± 0.19 mm. This MBL change did not differ significantly between the three prosthesis groups (*p* < 0.05) (Table 5).

**Table 5:** MBL of each prosthesis group at prosthetic loading and 5 year after implant installation

	Bridge (*n* = 13)	Hybrid denture (*n* = 10)	Overdenture (*n* = 6)
Prosthetic loading (T2)	0.52 ± 0.29	0.42 ± 0.21	0.60 ± 0.15
5 years after installation (T6)	1.08 ± 0.62	1.15 ± 0.34	1.2 ± 0.34
T6–T2*	0.60 ± 0.31	0.64 ± 0.36	0.58 ± 0.19

*The change between MBL at 5 years after installation and MBL at prosthetic loading did not differ significantly between the three prosthesis groups (*p* < 0.05)

## Discussion

ABGs are well-established and widely used in the reconstruction of edentulous jaws affected by severe atrophy [5]. Autogenous bone harvested from intraoral and/or extraoral sites are associated with reliable prognosis [20]. In cases with large bone defects, when intraoral bone harvest cannot provide sufficient bone graft volume, the use of extraoral donor sites becomes inevitable. Along with autogenous bone from other donor resources, bone from the iliac crest is used for augmentation in a wide spectrum of ABG cases. In this study, all included patients had a condition of severe bone defect due to previous osteomyelitis treatment, cancer ablation, or severe atrophy. The extremely low residual bone volume made the SDI placement is impossible. Another considered solution, alveolar distraction osteogenesis, also had many disadvantages in these cases due to multi-dimension deficit, therefore was not indicated. With the utilization of IBG, the augmented sites gained a generous bone volume, both vertical and horizontal dimension, which ease the implant installation and provide a comparable outcome.

Despite its advantages, onlay bone grafts from the iliac crest are associated with high bone resorption, which is highest during the early healing phase [5, 14]. While bone volume is known to decrease in general, vertical bone height resorption is well established as a major complication [14]. The high reported resorption rates are potential late complications and their effects on the survival rate of implants placed in grafted bone are being scrutinized.

In this study, the MRR after 5 years was 42.5%. This resorption rate is within the range of results reported by other authors. Johansson et al. [21] reported reductions of bone graft volume ranging from 47 to 49% in a clinical study of atrophic maxillae after 6 months of healing. Some authors have reported resorption rates ranging from 12 to 60% during follow-up from 1 to 5 years post-loading of implants [5]. The resorption rate reported in the present study is high, but the vertical bone gain after augmentation surgery was generous, and resorption occurred mostly during the first year after reconstruction. The resorption rates after 2 years, 3 years, and 5 years did not differ significantly, and these resorption rates are therefore predictable and acceptable. In the early healing period, when a graft is integrating and immobilizing at the recipient site, proliferating cells can penetrate the transplanted bone and the bone may become vascularized [22–24]; the dominant process is inflammation and seems to lead to bone resorption during the early period. The resorption rate at 5 years post-installation slightly decreased. This change could be the result of the lack of bone height data from patient no. 7 and the slight incorrection of the panoramic radiograph. However, there was no significant difference between resorption rate at 5 years and 3 years post-installation. A bigger sample with long-term follow-up will be needed to verify the finding.

Many studies have demonstrated the success of dental implant systems placed in iliac bone [25–27]. The survival rates of implants placed in the augmented bone range from 60 to 100%, with the majority of reported survival rates being at least 90% [5]. In this study, 1 of 29 implants failed, and the survival rate was 96.56%. MBL is a generally accepted parameter that is often used to evaluate long-term clinical results. A mean MBL of ≥ 1.5 mm in the first year and MBL of ≥ 0.2 mm per year after that are considered the threshold for implant success [28]. In this study, the mean MBL after the first year was 0.85 ± 0.44 mm, the mean cumulative MBL after 5 years was 1.12 ± 0.50 mm, and the MBL change each year was not greater than 0.2 mm. These MBL results are within the threshold indicating success [28]; however, longer-term follow-up is needed to verify our results.

In many reports, greater MBL is often observed after implant placement and the first year of function than the following years [29]. Adell et al. [29] reported that after the first loading year, the MBL decreased significantly to an average of only 0.1 mm. In our studies, the early MBL (from implant placement to early post-loading period) is also observed and recorded. The MBL difference from the previous time point at 3 months post-installation, prosthetic loading, and after 1-year post-installation differed significantly and peaked at 1-year post-installation. These data at 2 years, 3 years, and 5 years post-installation follow-up decreased to an average of 0.1–0.15 mm per year and did not differ significantly.

In this study, there were three groups regarding prosthetic indication, including bridge, hybrid denture, and overdenture. However, the MBL change at 5 years post-installation did not differ significantly between the three prosthetic groups (*p* < 0.05). Besides the prosthetic indication, other factors such as prostheses material, opposing dentition or prostheses, and position of installed implant were various. In the present study, the small sample size is a limitation to assess and evaluate the effect of these factors to the MBL. Therefore, further studies in larger samples of patients are warranted to validate our findings.

The etiology of greater MBL during healing and early of implant loading period is still investigated. Some authors suggested that bone loss may occur if the occlusal loading is excessive [30]. In our clinical experience, to prevent the early MBL, the occlusal loading control plays an important role. Unlike natural teeth, implant fixtures are osseointegrated to the bone without the periodontal ligament. The implants are more sensitive to the overload occlusal forces than natural teeth and more susceptible to the MBL at the early loading period when the implant-to-bone interface is immature. This suggested a well-controlled occlusal contact when the prosthetic is fabrication and delivery, which can reduce the overstress on the implant, and meanwhile provide a progressive occlusal force for the bone formation and maturation.

Controversy remains regarding whether implant placement should be performed immediately after graft placement, or if it should be delayed after bone grafting. Most previous studies reported better results for the two-stage than the one-stage approach [33]. The hypothesis is that after a period of bone healing and revascularization, integration of the implant will be more favorable and stable. Some other authors have suggested that one-stage surgery is preferable because it reduces the number of surgical interventions and healing time [31]. However, there have also been reports of the high and unpredictable rates of bone resorption for the one-stage approach [32], which can lead to poor primary implant stability and poor prosthetic orientation. Meticulous case evaluation is recommended before using this technique.

Different methods for the assessment of alveolar bone height have been commonly used in periodontal research and practice. Even though the cone beam computed tomography (CBCT) and intra-oral radiograph are considered as the gold standards for observation and measurement of the periodontal bone loss, the panoramic radiograph also was proved that it has a comparable accuracy, especially with the help of digital correcting and measuring software, along with the convenience and time-saving advantages. Persson et al.’s study was to assess the agreement between intra-oral and panoramic radiograph. According to the result of this study, intra-oral and panoramic radiograph readings are in great agreement [34]. In another research, Takeshita et al. evaluated the diagnostic accuracy of conventional periapical radiography taken with film holders Rinn and Han-Shin, digital periapical radiography with complementary metal-oxide semiconductor sensor (CMOS), panoramic radiography, and CBCT in the measurement of alveolar bone loss. The authors concluded that compared with the control measurements, only conventional periapical radiography using Han-Shin film holder showed significant lower differences, whereas the values of CBCT were the closest to the control method [35]. Therefore, the result reported using panoramic radiograph in this study is comparable and, in part, can substitute for the CBCT or intra-oral radiograph.

In case no.4, the patient has endured an old trauma with naso-maxillary fracture and fracture in the left mandibular body. After that, due to a bone defect in the left mandibular angle area, this area is re-fixation, after which the occlusion was similar to the status before the initial trauma. However, 1 year after hybrid implant prosthesis loading, in a follow-up recall, the patient was found to have developed a slight malocclusion. Slight occlusal adjustment was needed to treat the patient. He was free of symptoms and had unrestricted mandibular motion after that. In addition, in this patient, the augmentation and implant hybrid prosthesis were placed in both maxillary and mandibular. It is well established that the bone resorption rate of the maxilla is pretty higher in the mandible. Therefore, after prosthetic loading 1 year (the period between T2 and T4), the dramatical increasing of bone loss is explainable. After the first loading year, the resorption rate returns to the same rate as in other patients (Fig. 5).

Based on 10 years of experience, we here recommend treatment considerations and techniques to reduce bone resorption. The bone graft should be modeled for precise adaptation to the recipient site. A wax stent can be used to determine adequate graft size and contour. Cortical bone should be taken longitudinally and the thick side placed on the mesial surface of the mandible. An oversized graft should be harvested to maintain sufficient graft volume after the initial resorption phase. The recipient site should be perforated with a 1.0-mm round bur to increase the blood supply. Cancellous bone should be compressed and packed between the graft bone and the recipient site. The grafted bone block should be fixed firmly to the basal bone with titanium miniscrews. If the graft is not fixed and immobilized well, the strain on the newly forming tissues and resorptive areas of the graft will be too great to allow new bone formation. We used 1.5-mm or 2-mm round drills for countersinking. The schedule for implant installation should be 3 to 6 months after reconstructive surgery. Even though an extended healing period can result in more stable bone condition, rehabilitation of the implant and prosthesis is essential to preserve the grafted bone. Beside the osseointegration, the soft tissue integration is also essential for the long-term success of the implant and prosthesis. A soft tissue barrier, which is obtained by the adaption of attached tissue around the transmucosal implant structures, is important for establishing a stable peri-implant soft tissue as well as crestal bone. The implant surgeon can achieve this goal with a careful evaluation and meticulous strategy to maintain the existing attached gingival zone at the installation site formed after the bone graft healing period, by a proper flap design and soft tissue manipulation.

Along with implant monitoring and maintenance at the clinic, effective patient’s adaptation and self-hygiene of the prosthesis are also essential to ensure the longevity of the dental implant. The patient should be introduced to specific methods according to each type of prosthesis including fixed prosthesis, hybrid prosthesis, or overdenture. The effectiveness of oral hygiene in each patient should be re-evaluation at each follow-up visit to keep the implants free of peri-implant infection. If the patient has the poor oral hygiene, re-education and shorter waiting time between follow-up periods are required.

## Conclusion

In patients with atrophic jaws, sufficient long-term reconstruction can be achieved through a combination of IBGs and dental implant systems. Despite significant early stage vertical bone resorption, we obtained high success rates and stable peri-implant bone levels over the long term. Surgery and prosthodontics must be planned carefully to reduce bone resorption. Further studies in larger samples of patients and over longer-term follow-up are warranted to validate our findings.
